# Monoclonal Antibody Combinations that Present Synergistic Neutralizing Activity: A Platform for Next-Generation Anti-Toxin Drugs

**DOI:** 10.3390/toxins7061854

**Published:** 2015-05-29

**Authors:** Eran Diamant, Amram Torgeman, Eyal Ozeri, Ran Zichel

**Affiliations:** Department of Biotechnology, Israel Institute for Biological Research, Ness Ziona 7410001, Israel; E-Mails: erand@iibr.gov.il (E.D.); amit@iibr.gov.il (A.T.); eyalo@iibr.gov.il (E.O.)

**Keywords:** MAbs, oligoclonal, combination, neutralization, synergism, toxin

## Abstract

Monoclonal antibodies (MAbs) are among the fastest-growing therapeutics and are being developed for a broad range of indications, including the neutralization of toxins, bacteria and viruses. Nevertheless, MAbs potency is still relatively low when compared to conventional polyclonal Ab preparations. Moreover, the efficacy of an individual neutralizing MAb may significantly be hampered by the potential absence or modification of its target epitope in a mutant or subtype of the infectious agent. These limitations of individual neutralizing MAbs can be overcome by using oligoclonal combinations of several MAbs with different specificities to the target antigen. Studies conducted in our lab and by others show that such combined MAb preparation may present substantial synergy in its potency over the calculated additive potency of its individual MAb components. Moreover, oligoclonal preparation is expected to be better suited to compensating for reduced efficacy due to epitope variation. In this review, the synergistic neutralization properties of combined oligoclonal Ab preparations are described. The effect of Ab affinity, autologous Fc fraction, and targeting a critical number of epitopes, as well as the unexpected contribution of non-neutralizing clones to the synergistic neutralizing effect are presented and discussed.

## 1. Introduction

Since the emergence of hybridoma technology [1], the research and the development of monoclonal antibodies (MAbs) has rapidly progressed. MAbs, well-characterized individual full or partial immunoglobulin molecules, are currently being developed for a broad range of indications, from diagnostics and imaging to the treatment of medical conditions such as cancer and infectious diseases. In recent years, MAbs and related products have been the fastest growing class of therapeutic agents [2].

The use of antibodies as therapeutics was conceived long before the development of MAb technology; passive immunity, *i.e.*, the transfer of antibodies from the serum of an immunized person or animal to a non-immunized one, was first demonstrated more than 120 years ago in a pioneering experiment by Behring and Kitasato in which serum therapy protected against diphtheria [3]. Serum therapy was commonly used to treat infectious diseases until the discovery of chemotherapy in the form of sulfonamide in the 1930s [4]. Antibiotics were less expensive and difficult to use, had significantly fewer adverse effects, and were not pathogen-specific, rendering the diagnosis of the microbial infection unnecessary before treatment; thus, serum therapy was pushed aside. However, the emergence of multi-drug-resistant forms of new and old pathogens and the recent explosion of the immunocompromised population worldwide have led to a necessary resurgence in the development of antibody-based therapeutics to treat and prevent this new wave of drug-resistant infectious diseases [5]. Furthermore, whereas antimicrobial drugs can kill the microbes but cannot eradicate pre-formed toxins, a specific antibody is the only compound that can neutralize a given toxin [3]. Therefore, antibodies remain attractive for therapeutic purposes.

However, modern polyclonal antibody (PAb)-based therapeutics, although improved compared with past serum therapies, continue to suffer from major limitations that might be elegantly overcome by MAb-based preparations. First, treatments with hyperimmune equine- or other animal-derived antisera are associated with substantial side effects, including hypersensitivity-related reactions such as serum sickness and anaphylaxis [6,7]; in addition, the use of human antisera involves the risk of blood-borne disease [8]. These safety limitations can be addressed by using human-derived or humanized MAb-based preparations that display a substantially decreased incidence of side effects and viral contamination [9,10]. Humanization, the replacement of mouse constant regions and variable framework with human sequences, results in a product displaying significantly reduced immunogenicity and improved *in vivo* tolerability [11]. Human or humanized MAbs exhibiting enhanced pharmacokinetics enable the administration of a lower protein load and a reduced frequency of administration during the course of treatment. Second, PAb-based preparations exhibit significant batch-to-batch variability, and their supply is limited. In contrast, MAbs can be produced *in vitro*, thereby ensuring an unlimited supply of highly purified, well-characterized products that are devoid of contaminating proteins [8].

Despite the potential of MAbs as powerful tools in the fields of infectious diseases and toxins, during the past 25 years, following the hybridoma revolution, out of the more than 30 immunoglobulins (IgGs) and their derivatives that have been approved for use for various indications [2], only one MAb has been approved for the prevention of a viral infection (RSV) [12], and one MAb has been approved in the U.S. for the treatment of bacterial toxin (anthrax) [13]. All others were clinically designed for the treatment of cancer and autoimmune or allergic conditions [14]. Although many MAbs have been purified and characterized for their protective efficacy against different toxins [3], some of which are under investigation in clinical trials [7,15], PAbs remain almost the only available preparations for this indication. MAb-based therapy has some limitations. First, MAbs exhibit unprecedented specificity to their antigenic target, but this extreme specificity may hamper the efficacy of any individual MAb in the case of the absence or modification of its target epitope in a mutant or subtype of the infectious agent. Second, the potency of MAbs, especially those that play a role in the neutralization of pathogens and toxins, remains relatively low compared with PAb-based preparations. This inferior neutralizing potency of individual MAbs may be attributed to the differences in the functional impact of specific antigenic epitopes, to their low affinity to the target epitope, to the biochemical stability of each MAb molecule, or reduced clearance of the MAb-antigen immune-complexes (ICs). These two major limitations of individual MAb-based preparations, their low neutralizing potency and potential failure to treat mutants or subtypes, can be overcome by combining several MAbs to form a MAb cocktail. Moreover, as will be further discussed in details, recent studies show that such MAb cocktails exhibit synergistic neutralization; *i.e.*, MAb cocktails display substantially elevated neutralizing activity that exceeds the level of improvement expected by the contribution of each MAb in the mixture. In fact, the neutralizing activity of MAb cocktails can be similar to the potency of PAb-based preparations.

This phenomenon was demonstrated in our lab as well. We generated MAbs against botulinum neurotoxins (BoNTs). We not only simultaneously obtained neutralizing MAbs against different BoNTs in a single automated process but also successfully demonstrated significant synergistic neutralization by the combined anti-BoNT MAbs [16]. BoNTs, which serve as an excellent model for complex antigens against which MAbs are required, are produced by Clostridium botulinum strains and are considered the most lethal toxins identified, with an estimated human median lethal dose (HLD_50_) of 1 ng/kg body weight [17,18]. BoNTs are the only toxins classified by the CDC as category A agents [19]. However, the standard treatment for botulism relies on equine or limited amounts of human PAb-based antitoxin therapy, together with supportive care [20]. Thus, there is a need for safe and reliable anti-BoNT MAb-based pharmaceutical drugs.

In this review, we describe the mechanisms underlying the neutralizing synergy of oligoclonal-based preparations, with an emphasis on biological toxins. The importance of Ab affinity, the molecular structure of IgGs involved in MAb-antigen ICs clearance, and the unexpected contribution of individual non-neutralizing MAbs to the synergistic neutralizing effect are discussed.

## 2. Synergistic Neutralization of MAb Cocktails

Natural human polyclonal responses are elicited by the concerted action of antibodies that display multiple specificities and bind to several epitopes. Using carefully assembled cocktails consisting of recombinant human MAbs, it might be expected that these mechanisms would be more efficiently recruited than using single MAbs [21].

The concept of combining several MAbs to increase their therapeutic efficacy and to overcome the limitations of PAbs appears to be very logical. Indeed, in the last 30 years, many studies have evaluated two or more MAbs in various combinations to improve the efficacy of these potential therapeutics for infectious disease, primarily viruses and toxins (see Table 1 for a list of oligoclonal antibody-based preparations against various toxins). Most of these MAb combinations exerted additive or synergistic neutralizing effects, demonstrating their potential value as future therapies.

**Table 1:** The neutralizing effect of MAb combinations.

Toxin	MAb number ^a^	Fold enhancement ^b^	*In vivo*/*in vitro*	Reference
Botulinum A	3	20,000	*in vivo*	[22]
	2–4	100	*in vivo*	[8]
	2–5	10,000 ^c^	*in vivo*	[23]
	2–4	>100	*in vivo*	[24]
	2	<10	*in vivo*	[25]
	2	1	*in vitro*	[26]
	2	40	*in vivo*	[27]
	7	150	*in vivo*	[16]
	2 ^d^	166	*in vivo*	[28]
Botulinum B	2–4	No ^e^	*in vivo*	[29]
	2–3	30	*in vivo*	[30]
	2–7	10	*in vivo*	[16]
Botulinum E	8	400	*in vivo*	[16]
Difficile A	2	~2	*in vitro*	[31]
Ricin	2	1.5	*in vitro*	[32]
	3	7.5 ^f^	*in vivo*	[33]
	2	~2	*in vitro*	[34]
Pertussis toxin	2–3	1	*in vivo*	[35]
	2	No ^e^	*in vivo*	[36]
	2	>10	*in vivo*	[37]
	2	4	*in vitro*	[38]
	2	>1.5	*in vivo*	[39]
Anthrax	2–3	10–100	*in vivo*	[40]
	2	1	*in vitro*	[41]
	2	1	*in vivo*	[42]
	2	~10	*in vivo*	[43]
	2	7	*in vivo*	[44]
	3	Delay death	*in vivo*	[45]
	2	>2 ^g^	*in vitro*	[46]
	2	~10	*in vivo*	[47]
	2	1.7, 3.8 ^h^	*in vitro*	[48]
SEB	2	<10	*in vivo*	[49]
	2	>10	*in vivo*	[50]
Tetanus	2–4	20	*in vivo*	[51]
	2	<10	*in vivo*	[52]
	2	3	*in vivo*	[53]
PLY	2–3	>5	*in vivo*	[54]
Scorpion Aha venom	2	<10	*in vivo*	[55]

^a^: Number of MAbs in the evaluated cocktail. ^b^: Values are estimated as indicated in the text. A value of 1 indicates an additive effect. ^c^: Mice treated with MAb combinations survived 10,000 MsLD_50_, whereas the individual MAbs were not protective. ^d^: Using 2 HPs. ^e^: Neither of the MAbs alone nor in combination had neutralizing activity. ^f^: The combination of three MAbs enabled protection of the mice 7.5 h after exposure compared with 1 h for each MAb alone. ^g^: Dependent on the type of cell line used (J774: Synergism. CHO: Additive effect). ^h^: DRI for each MAb.

When combining two or more drugs, such as anti-toxin MAbs, four outcomes can be expected: an additive effect, *i.e.*, the summation of all individual neutralizing activities; antagonism, defined as a reduction in efficacy; indifference, *i.e.*, one drug does not enhance or reduce the efficacy of the second drug; and synergism, in which the protective properties of the combined MAb preparation significantly exceed the additive effect of the components in the mixture.

Attempts to estimate the combined effect of multiple drugs have been documented for more than a century [56]. Chou and Talalay proposed a general diagnostic function to determine the applicability of experimental data and to distinguish between mutually exclusive and nonexclusive drugs and provided equations for analyzing and quantifying the synergism, summation and antagonism of multiple drugs [56]. The authors designated a “combination index” (CI), in which a value of <1, =1, or >1 indicates synergism, an additive effect (summation) or antagonism, respectively, for each drug combination. Similarly, Pohl *et al.* applied the fractional inhibitory concentration (FIC) indices approach for the evaluation of drug combinations to characterize the interactions among multiple MAbs; similar to the CI, the interaction between two MAbs is considered to be synergistic if the FIC index is <1.0, additive if the FIC index is equal to 1, indifferent if the FIC index is between 1 and 2, and antagonistic if the FIC index is >2 [47].

A synergistic drug combination should facilitate the reduction of drug doses while maintaining efficacy. The fold-reduction in the dose of each drug included in a synergistic combination at a given effect level, compared with the dose of each drug alone, can be calculated as the dose reduction index (DRI) [48]. The CI and the DRI also enable a useful comparison of the extent of efficacy enhancement between neutralizing MAb combinations in different studies. Although many of the studies involving MAb cocktails do not provide direct data regarding these values, in most of these studies (Table 1), an enhancement in the neutralization of MAb combinations was detected compared with that of the individual MAb components. This enhancement is often referred to as ‘synergism’ by authors; however, although enhanced neutralizing effects are reported, it is difficult to compare the extent of this synergy among different studies due to various factors, including toxin biology and the nature of the neutralization assay, which can be conducted either *in vivo* (for instance, a mouse protection assay) or *in vitro* (cell toxicity assays, using different cell types). Thus, the information in the table was estimated after careful analysis in light of this ‘fold-enhancement’ (the terminology that we selected to describe the superiority of a MAb combination compared with its components). In some cases, inferring the difference in neutralization was not applicable.

In a previous study conducted by our lab, specific monoclonal antibodies against the three botulinum serotypes A, B and E were generated by immunizing mice with a trivalent mixture of the recombinant *C*-terminal half of the heavy chain (Hc) of botulinum neurotoxins A, B, and E [16,57].

Following immunization, we conducted parallel differential robotic hybridoma screening to identify specific MAbs against each of the three toxins. The integration of automated robotic liquid-handling systems can significantly improve the overall screening capacity of hybridoma [10].

**Figure 1:**
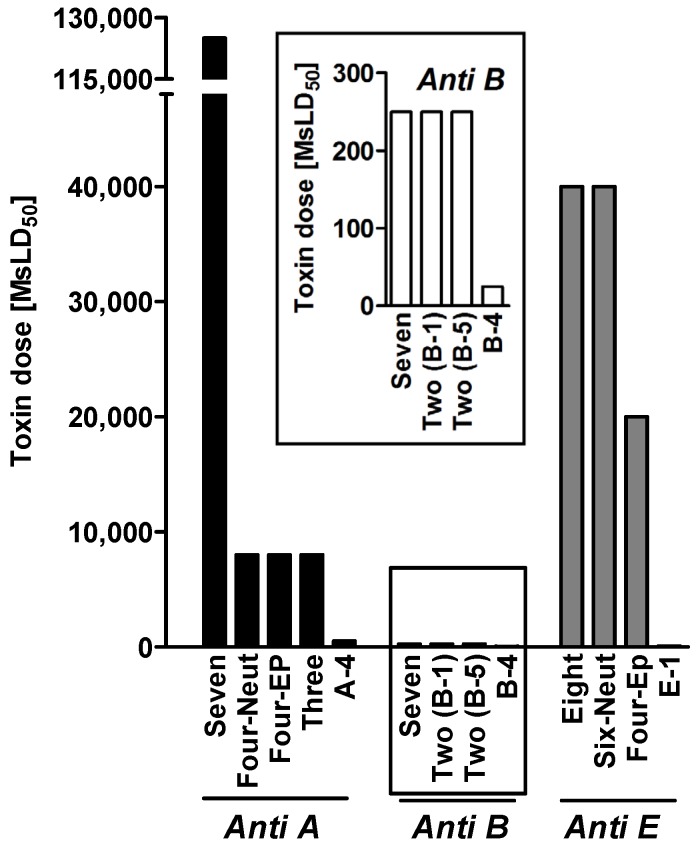
Neutralizing activity of oligoclonal combinations [16]. Different BoNT doses were pre-incubated with combinations of equally diluted MAb ascites fluids (final dilution 1:200, equals ~25 µg/mL of IgG) and then injected into mice. The results indicate the maximal toxin dose that the mice could withstand. Anti-serotype B MAb results are zoomed separately. Anti-serotype A MAb panel: Seven [A-4, A-1, A-6, A-2, A-3, A-8, A-7], Four-EP [epitope recognition recognition-based MAbs A-4, A-1, A-3, and A-8], Four-Neut [neutralizing MAbs A-4, A-1, A-6, and A-2], Three [A-4, A-1, and A-8 or A-3]. Anti-serotype B MAb panel: Seven [B-4, B-2, B-1, B-3, B-6, B-5, and B-7], Two (B-1) [B-4 and B-1], and Two (B-5) [B-4 and B-5]. Anti-serotype E MAb panel: Eight [E-2, E-3, E-4, E-5, E-6, E-7, E-8, and E-1], Six-Neut [neutralizing MAbs E-2, E-3, E-4, E-5, E-7, and E-1], Four-EP [epitope recognition-based MAbs E-2, E-3, E-8, and E-1], and E-1.

Within each serotype-specific group, we produced neutralizing MAbs, the majority of which were protective against a toxin dose of 10 Mouse Lethal Dose 50% (MsLD_50_). Encouraged by these results, we sought to determine whether the MAb combinations in each serotype-specific group exhibited increased neutralizing activity. The best combination for anti-serotype E MAbs displayed a very high synergistic neutralizing activity of up to 400-fold over that of its components (Figure 1). The cocktail of anti-serotype A MAbs, which protected against 125,000 MsLD_50_ of toxin A, had a 154-fold improvement over the calculated additive effect of the mixture’s components.

Strikingly, the removal of three non-neutralizing MAbs from the 7-clonal anti-BoNT/A cocktail dramatically reduced the neutralizing activity. The importance of non-neutralizing individual MAbs was also demonstrated for anti-B MAbs, confirming that addition of a single non-neutralizing MAb to the neutralizing antibodies may induce synergism.

Others also found that a combination of seven apparently non-neutralizing MAbs with one neutralizing MAb directed to the heavy chain of the tetanus neurotoxin (TeNT) induced synergistic neutralization [51].

The induction of synergistic neutralizing effects by a combination of non-neutralizing MAbs is critical to our comprehension of the mechanisms underlying synergism and may have important consequences for the future screening and selection of potent neutralizing MAb cocktails. What are the mechanisms underlying this antibody-mediated synergy? Toxin neutralization depends, at least in part, on functional “neutralizing” epitopes recognized by antibodies that interfere with the interaction of the toxin with the target cell, on the binding affinities of the antibodies, and on the quality of antibody-toxin complex clearance from the bloodstream. Accordingly, the induction of neutralizing synergism might be explained by at least three putative mechanisms: first, the simultaneous binding of interfering MAbs to multiple functional sites on the toxin; second, an increase in the affinity of the mixture of MAbs compared with each MAb component; and third, enhanced Fc-mediated clearance of antibody-toxin complexes from the serum due to multimeric antibody decoration of the toxin. Each of these three mechanistic explanations for this synergy may exert a partial influence in different MAb combinations and may be considered a different aspect of a tri-partite phenomenon; together, these three mechanisms may induce synergistic neutralization.

### 2.1. Simultaneous Binding of Interfering MAbs to Multiple Non-Overlapping Functional Epitopes

The humoral response to evading pathogens involves a series of effector mechanisms of antibodies that include direct binding to specific epitopes and indirect antibody-mediated events primarily consisting of antibody-dependent cellular cytotoxicity (ADCC), antibody-dependent cell phagocytosis (ADCP), and complement-dependent cytotoxicity (CDC) [58]. For more than a century, toxins have been believed to be neutralized by antibodies not by indirect mediated measures but rather by a direct “interfering” antibody-dependent mechanism in which antibodies bind to toxins and interfere with their interactions with host cells, thus preventing the attachment of the toxin to the target cell or its enzymatic activity that might otherwise damage the target cell. Theoretically, for an antibody to interfere with a toxin, only the variable region of the antibody is required because it is the moiety that specifically recognizes and binds to its epitope on the target toxin. Thus, this binding alone is generally considered as “neutralization”. The constant region (Fc) of an antibody is known to exert effector functions (ADCC, ADCP, CDC) and is thought to become activated by bacteria and tumor cells [2,14], but the involvement of these mechanisms in humoral anti-toxin responses is unclear. Alternative roles of Fc in the clearance of ICs and in antibody pharmacokinetics are discussed in Section 2.3.

#### 2.1.1. Functional Neutralizing Epitopes

Toxin neutralization has been considered to be primarily due to the binding of the Ab to the toxin, blocking its activity [59]. To design a combination of MAbs that mimic a polyclonal response, the principal rationale would be to select MAbs that bind to distinct or non-overlapping epitopes. Such a combination may facilitate the binding of multiple IgG molecules to the target toxin, thus potentially blocking various essential functional domains. This simultaneous blockade increases the probability that these MAbs will interfere with interactions between the toxin and its target cell, ultimately resulting in either additive or synergistic neutralization.

It has been demonstrated that particular domains on different toxins are associated with enhanced neutralization, depending on their structure and mechanism of action. For instance, Hc (50 kDa) is considered to be the receptor binding domain of the BoNT to the target neuron, whereas the N-terminal region (Hn) serves as the translocation domain; the catalytic domain is a zinc endopeptidase that is localized to the light chain (L). The surface residues of Hc vary dramatically among different BoNT serotypes [60]. The Hc fragment itself is non-toxic, but most neutralizing epitopes have been mapped to this domain [61], and the ‘neutralizing’ epitope-rich Hc has been used as a subunit vaccine to produce neutralizing antibodies against BoNT [57,62].

Bacillus anthracis, the causative agent of anthrax, exerts its toxicity via the dissemination of a tripartite exotoxin composed of protective antigen (PA), lethal factor (LF) and edema factor (EF). PA plays a critical role in anthrax pathogenesis, as it associates either with LF to form the lethal toxin (LeTx) or with EF to form the edema toxin (EdTx) [48]. Vaccines composed of PA showed that PA-binding antibodies effectively limit B. anthracis pathogenicity [63]. Moreover, it was demonstrated both for PA and for LF that binding of specific protective epitopes is required for neutralization [47,64,65,66].

Pertussis is primarily a toxin-mediated disease caused by Bordetella pertussis. Pertussis toxin (PTx) is a major virulence factor, and chemically or genetically detoxified PTx is a major component of all acellular vaccine formulations in combination with up to four additional virulence factors. It has been shown that PTx-neutralizing Abs bind to its S1 and S2/3 subunits, which are necessary for its translocation to the cytoplasm and endocytosis, respectively [67].

Ricin (molecular weight, 64,000) is a relatively simple toxin consisting of an enzymatic A subunit (RTA) and a binding B subunit (RTB) joined by a disulfide bond [68]. O’Hara *et al.* found that epitope specificity is the primary determinant of the ability of an antibody to neutralize ricin. These specific epitopes interfere with the enzymatic activity of RTA. Moreover, neutralizing MAbs were primarily directed against α-helices situated within RTA folding domains 1 and 2, whereas non-neutralizing MAbs targeted random loops and coils largely localized to domain 3 [69].

In rare cases, a single MAb can be as protective as a PAb-based preparation [53,70] via the blockade of a pivotal epitope that results in complete neutralization. This evidence for the existence of ‘hot spot’ epitopes that display a unique functional impact suggests that interfering with more than one such epitope might be highly beneficial for toxin neutralization.

#### 2.1.2. Simultaneous Interference with Multiple Functional Epitopes

For most of the reported neutralizing MAbs, the potency of an individual MAb is substantially lower than that of a PAb. Various studies have reported that the potency of protective MAbs can be augmented additively or synergistically via the addition of other protective MAbs and that the combination of protective MAbs targeting different epitopes on a toxin molecule can synergize protective efficacy [71]. However, the exact mechanism underlying synergistic rather than merely additive neutralization following the blockade of multiple significant epitopes is not fully understood.

**Figure 2:**
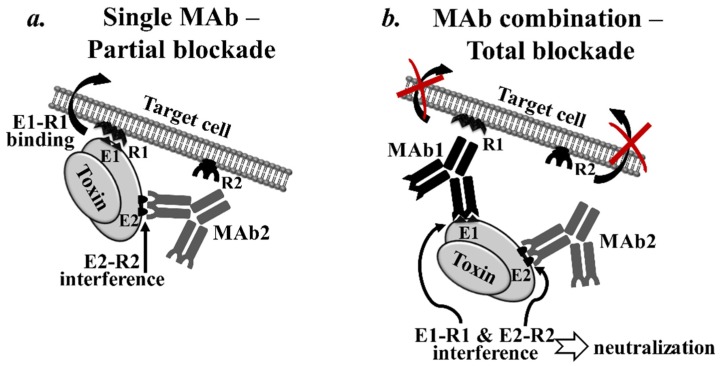
Schematic illustration of the simultaneous interference with multiple functional epitopes. Synergistic neutralizing effect may be the result of antibody interference with two or more distinct epitopes that, although not essential individually (**a**), are vital in combination (**b**). Important functional pathways of the toxin, such as binding to its receptor target, might be eliminated by this concurrent blockade. Abbreviations used: E—epitope, R—receptor on a target cell.

Such synergy may stem from interference with two or more epitopes that, although not essential individually, are vital in combination. The concurrent blockade of these types of epitopes might synergistically “shut down” essential functional pathways of the toxin, such as binding to its receptor target. This phenomenon is expected to be more prominent when important neutralizing epitopes perform redundant functions, such as binding to cellular receptors, cleaving a target substrate, *etc.* (Figure 2). The following findings support this possibility.

For BoNTs, two classes of binding sites exist, proteins and ganglioside receptors, both of which reside in the Hc domain. Thus, a double-receptor concept is prevalent [72]. In a study in 2012, Chen *et al.* predicted that two MAbs, which recognized the protein and the ganglioside receptor binding domains, respectively, synergistically neutralized BoNTs. In this study, the investigators selected three neutralizing MAbs that recognized different non-overlapping epitopes of BoNT/B and compared the neutralizing effects of various antibody combinations. They found that a MAb that responded to the ganglioside receptor binding site synergized with the other two MAbs that recognized non-overlapping epitopes in Syt II (the protein receptor). However, the combination of the latter two MAbs did not exert a synergistic effect [30]. This example demonstrates that the combination of two MAbs that recognize distinct functional domains on BoNTs may represent the general principle for a potent synergistic effect. Interestingly, Chen *et al.* further analyzed the results generated by the lab of James Marks [22], which developed MAbs for BoNT/A. They found that the neutralizing potency of one pair of MAbs (C25 + 3D12), which bind to the translocation domain and the sialoganglioside binding domain, respectively, was 10 times higher than that of another pair (S25 + 3D12), whose epitopes are located at the same functional domain. This finding provides additional evidence that a combination of MAbs that recognize different functional domains is more likely to synergize than a combination of MAbs recognizing the same functional domain [30].

The RTB subunit of ricin contains two galactose binding sites that function independently and are distant from one another [34,73]. Hu *et al.* generated four MAbs directed against the RTB subunit and found that all of them bound to conformational epitopes with a high affinity. When testing pairs of MAbs, they demonstrated a synergistic neutralization of ricin *in vitro* and *in vivo*. Hu *et al.* hypothesized that because the two RTB domains are homologous and are structurally similar, a single RTB-specific MAb may block both galactose binding pockets by binding to similar conformational epitopes sharing both domains and that the simultaneous binding of two MAbs likely synergistically interferes with ricin attachment to cells. In addition, the authors speculated that MAbs might bind to domains that interrupt the transport of RTA from the ER to the cytosol. They also determined that the synergistic effect of the MAb pairs depended on different ricin neutralization activities that were more related with their epitope specificities than their ricin-binding affinities and that were not related to their antibody isotypes [34]. In another ricin study, McGuiness *et al.* identified and characterized a murine IgG1 anti-RTB MAb (24B11). Using a Vero cell cytotoxicity assay, they compared 24B11 with a known anti-RTA MAb (R70) and found that 24B11 was approximately two-fold more effective at neutralizing ricin. The investigators studied the mechanism of action of these two MAbs and found that both 24B11 and R70 blocked ricin attachment to galactoside receptors, although R70 was directed against RTA [32].

Active TeNT, a close relative of BoNT, is a di-chain toxin, in which the light chain (LC) and heavy chain (HC) are linked by a single disulfide bond. The 50 kDa LC acts as a zinc-dependent endopeptidase, and the 100 kDa HC is composed of two distinct functional domains: A translocation domain (*N*-terminal half) and neuronal ganglioside binding domain (*C*-terminal half) [60]. Similar to BoNT, it is accepted that TeNT acts via a dual receptor model in which gangliosides and glycosylated proteins, such as the synaptic vesicle proteins SV2A and SV2B, are involved [74]. In one pioneering study of MAb combinations against toxins, Volk *et al.* evaluated the effect of MAbs on different TeNT subunits and found that all neutralizing MAbs bound to epitopes on the HC of TeNT (approximately half of them to the *C*-terminal region and half to the *N*-terminal region). When combining these MAbs, it was found that different cocktails of 2–4 MAbs exerted synergistic neutralization *in vivo*, resulting in up to 200-fold greater protection than the individual neutralizing MAbs. The authors hypothesized that the large number of epitopes found to be involved in neutralization suggested that different antibodies block distinct steps in toxin function and that the simultaneous binding of multiple epitopes is required for such neutralization [51].

Synergistic oligoclonal antibody interference may not necessarily be associated with known neutralizing epitopes but may rather be based on either adherence to epitopes that individually play no apparent role but together perform a significant function or the blockade of functional epitopes due to steric hindrance caused by the valence of a nearby bound antibody. For instance, Cheng *et al.* demonstrated that a combination of two MAbs, one directed against the LC (catalytic domain) of BoNT/A, and the other directed against the translocation domain, did not inhibit endopeptidase activity but, instead, blocked toxin entry into primary and cultured neuronal cells in a synergistic manner. The authors suggested that, via steric hindrance, MAbs may have interfered with BoNT/A receptor binding, subsequent neuron internalization, or interaction with intracellular SNAP25 [25].

Similarly, synergistic effects of the combination of protective and non-protective MAbs were demonstrated for a pair of a protective MAb and a disease-enhancing MAb against anthrax by the lab of Arturo Casadevall. Chow *et al.* investigated whether the combination of a protective and a disease-enhancing MAbs act in concert to abrogate PA oligomerization, thus preventing the translocation of LF and EF into the cell to cause subsequent toxicity. Individually, the protective MAb inhibited furin cleavage and subsequent oligomer formation, whereas the enhancing MAb did the opposite. However, the IgG MAb combination resulted in MAb-PA complex formation that completely abolished PA oligomerization via a furin cleavage-independent mechanism [71].

Evidence supporting the concept that synergistic activity is partially derived from the combination of MAbs that simultaneously block different functional epitopes was provided by the neutralization of Staphylococcal enterotoxin B (SEB), one of several potent exotoxins secreted by Staphylococcus aureus that cause toxic shock syndrome (TSS) [75]. Similar to other superantigens, SEB simultaneously binds to major histocompatibility complex class II (MHC-II) molecules on antigen-presenting cells (APCs) and to T-cell receptors (TCRs) that incorporate V chains [50]. Blocking antibodies may prevent SEB from facilitating the formation of the MHC-II/SEB/TCR complex, thereby inhibiting this toxin. According to the crystal structures of complexes consisting of SEB and MHC-II or TCR, the two binding sites are spatially distinct from the contact areas [76]. Varshney *et al.* generated MAbs against SEB and evaluated different pairs of MAbs in which only one or none of the MAbs individually protected against SEB toxicity. The authors reported that protection was achieved when these MAb combinations were administered simultaneously. In fact, this was the first report in which enhanced protection against SEB-induced lethal shock (SEBILS) was demonstrated in mice by the combination of two individually non-neutralizing MAbs. These MAbs recognized non-continuous residues that likely contribute to conformational epitopes [50].

The phenomenon of synergistic neutralization due to blockade of functional non-overlapping epitopes is not restricted to toxins, and has also been demonstrated in the study of viruses and bacteria. Li *et al.* evaluated different combinations of human MAbs directed against various epitopes on human immunodeficiency virus type 1 (HIV-1) envelope glycoproteins for their ability to neutralize a chimeric simian-human immunodeficiency virus (SHIV-vpu1). In accordance with the putative synergistic mechanism, the most potent neutralization of triple combinations evaluated was attained using three MAbs directed against three different domains, one each on V3, gp41, and gp120. The authors further evaluated a combination of four MAbs (the previous three MAbs and one that recognized the CD4 binding site) and found higher neutralization potency and a higher degree of synergy than any of the triple combinations [77]. Similarly, Meulen *et al.* presented experimental animal data showing that protection against severe acute respiratory syndrome coronavirus (SARS-CoV) infection was feasible using a mixture of two human MAbs (CR3014 and CR3022). This MAb combination neutralized SARS-CoV in a synergistic manner by recognizing different epitopes on the receptor-binding domain. [78].

Faleri *et al.* recently reported the functional characterization of two MAbs that recognize Factor H binding protein (fHBP) on Neisseria meningitidis serogroup B (MenB) surface [79]. fHBP has proved to be a virulence factor for N. meningitidis and a target for functional bactericidal antibodies. fHBP binds to human factor H (hfH), that serves to downregulate the host alternative complement pathway, and helps the organism evade host innate immunity [80]. Although the MAbs recognized two overlapping epitopes that were part of the hFH binding site on the N-terminus of the hFBP, they did not elicit complement mediated bactericidal activity against MenB, when tested either individually or in combination. Interestingly, when tested in combination with another MAb, that recognized a conformational epitope located on the *C*-terminus—opposite to the hFH binding site, the two MAb combinations exerted different levels of synergistic activity, depending on the orientation of the MAbs on fHBP. The authors suspected that while the concomitant binding of one combination may result in a reciprocal orientation suitable for correct engagement of C1q, the relative position of the other combination might not be optimal to promote the same effect [79].

In summary, combining MAbs that bind to functionally distinct epitopes of a toxin may result in synergistic neutralization. Moreover, even the combination of MAbs that individually do not display neutralizing activity may exert a synergistic neutralizing effect due to either the steric hindrance of adjacent neutralizing epitopes or partial or full epitope redundancy that is overcome by simultaneous binding.

### 2.2. Increase in Affinity of MAb Cocktails Targeting Multiple Epitopes

Another mechanism that may underlie the synergistic neutralizing activity of a MAb combination is the increased affinity of the cocktail compared with each MAb component. Various findings indicate that neutralization potency is associated with the affinity of the antibody to the toxin. As demonstrated in the following section, additional findings have indicated that the simultaneous binding of multiple MAbs to a multisite antigen may result in a substantial increase in overall affinity. Taken together, these findings suggest that certain combinations of MAbs bind to a toxin much more strongly than each MAb alone, thus inducing synergistic neutralization.

#### 2.2.1. Role of Affinity in Toxin Neutralization

The correlation between antibody binding affinity and toxin neutralization is somewhat intuitive, as toxin blocking and the subsequent clearance of the formed ICs depend on the binding of antibodies to the toxin. Thus, one can easily conceive that adherence to the toxin would be more efficacious if the binding of the antibody to its epitope were stronger, thus prolonging the duration that this epitope is ‘occupied’, leading to more potent inhibition and clearance from the bloodstream. There are several indications that the neutralizing activity of antibodies is associated with their affinity to the target epitope. Indeed, *in vivo* assays conducted in our lab revealed that the neutralizing potency of MAbs against the three different BoNTs was associated with an increased affinity to the toxin, as all neutralizing MAbs exhibited the highest anti-toxin ELISA titer [16]. Pless *et al.* also reported that a common characteristic of all anti-BoNT/A-Hc neutralizing MAbs examined was a very high affinity [81]. In another anti-BoNT study, Amersdorfer *et al.* showed that the most potent anti-Hc scFv displayed the highest affinity for its epitope [82]. In accordance with this concept, Maynard *et al.* showed that the protection against anthrax toxin by recombinant antibody fragments was correlated with antigen affinity [83]. Likewise, Scott characterized a panel of anti-tetanus toxin scFvs and found that 14/15 clones neutralized toxin activity based on a ganglioside binding assay and that this effect was strongly related to the affinity of these clones [84].

Nevertheless, we found that some MAbs exhibited no neutralizing activity despite displaying a high ELISA titer. These results suggest that high affinity may be a necessary but not sufficient parameter of neutralizing potency [16].

#### 2.2.2. Synergistic Neutralization Due to Enhanced Affinity

The findings described in the previous section demonstrate that the affinity of an antibody to its epitope correlates to its neutralizing activity. Thus, if the simultaneous binding of multiple MAbs increases the overall affinity of the MAbs to the antigen, the neutralizing activity of the combined MAbs may be enhanced.

What is ‘overall affinity’, and how can it be increased? Affinity, a measure of the strength of the noncovalent interaction between an antibody and its antigen [85], is mathematically represented by the equilibrium constant (K) that characterizes the reversible binding of an antibody to an epitope on an antigen [86]. Affinity can be divided into ‘intrinsic’ and ‘functional’. The term ‘intrinsic affinity’ characterizes systems involving monovalent interactions between one paratope and one epitope, while the term ‘functional affinity’, also known as ‘avidity’, refers to higher order complexes between bivalent or multivalent Abs and a number of binding sites per antigen [87], and reflects the intrinsic affinity, the valence and the topological relationship between the antigenic determinants and the antibody combining sites [88]. The strength of multivalent interactions (functional affinity or avidity), usually exceeds, sometimes greatly, the corresponding intrinsic affinity [85,86]. Multivalent linkages within ICs can be formed between either the same epitope expressed on separate antigens and Ab molecules of the same specificity (MAb), or between diverse epitopes and Abs with two or more specificities (such as in the case of natural immune reactions), which may have cooperative interactions between them, eventually leading to increase in the functional affinity.

**Figure 3:**
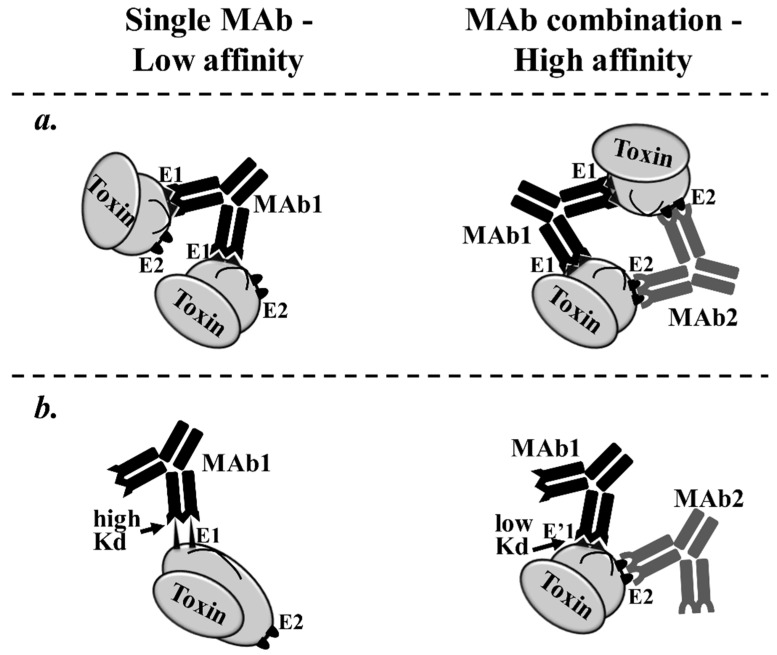
Schematic illustration of the increase in affinity of MAb cocktails targeting multiple epitopes. The binding of multiple MAbs to a multisite antigen may result in a substantial increase in overall affinity via two basic mechanisms. (**a**) Upper panel: The binding of anti-E1 MAb to E1 epitopes on two soluble antigens facilitates their cross-linking, thereby increases the probability that an anti-E2 MAb would bind to two sites instead of to only one site. (**b**) Lower panel: A conformational change in the antigen upon the binding of anti-E2 MAb that thermodynamically favors the binding of ananti-E1 MAb. Thus, in the presence of MAb2, MAb1 binds E’1 with a substantial lower Kd. E1—epitope 1, E’1—epitope 1 following a conformational change of the toxin as a result of MAb2 adherence to epitope 2 (E2).

Two basic mechanisms may underlie such cooperative interactions between MAbs that bind to diverse epitopes. First, the binding of one bivalent MAb to two epitopes on two soluble antigens, thereby facilitating their cross-linking, increases the probability that a second MAb, specific for an independent epitope, could bind to two sites instead of to only one site [85]. This phenomenon was demonstrated 30 years ago by Moyle *et al.* for antibodies against human chorionic gonadotropin (hCG). The authors found that some MAb pairs but not others displayed a higher affinity to the hormone than either MAb alone and that this increased affinity was associated with the formation of a stable circular complex consisting of two antibody molecules (each one of the two MAbs) and two hCG molecules [89]. Second, the increase in functional affinity observed for multiple MAbs may be due to a conformational change in the antigen that occurs upon the binding of the first MAb, resulting in a higher affinity of the second and third MAbs [22,85] (illustrated in Figure 3). Thus, the binding of the first antibody might induce a more stable conformation of the antigen that thermodynamically or entropically favors the binding of a subsequent antibody [84,85].

In a comprehensive study conducted by the lab of James Marks, Nowakowski *et al.* elegantly linked synergistic toxin neutralization to an enhanced affinity of MAbs to BoNT/A HC and demonstrated that although no single MAb effectively neutralized the toxin, combinations of three MAbs resulted in significant neutralization both *in vivo* and *in vitro*. Significant synergy in toxin neutralization was detected for pairs of MAbs compared with each individual MAb *in vitro* (mouse hemidiaphragm assay) based on a significant increase in the time to neuroparalysis. The authors further showed that a large increase in the affinity of the MAb cocktail likely resulted in increased toxin neutralization potency. This result was demonstrated by determining the functional binding affinities for each single MAb, each pair of MAbs, and the mixture of all three MAbs. Strikingly, when one MAb (C25) was mixed with a second MAb (3D12), the resulting affinity of the MAb combination was 200- and 10-fold higher than the individual MAbs alone, respectively. The equimolar cocktail of all three MAbs (addition of MAb S25 to the other two) further increased the affinity by 4-fold. Interestingly, the affinity of the pair of MAbs C25 and S25 was only 2-fold higher that the single MAb affinities, and this pair was less potent *in vivo* compared with the other pair consisting of MAbs C25 and 3D12. Moreover, the magnitude of the increase in the *in vivo* neutralization potency of the different MAb combinations paralleled the increase in functional affinity. Thus, the neutralization potency of individual MAbs and mixtures of MAbs could be predicted based on the equilibrium binding equation according to the affinity data alone. The authors reported a synergistic effect resulting in a more than 20,000-fold increase in potency for the three-MAb combination compared with any individual MAb [22].

Evidence supporting the influence of affinity on the synergistic neutralization of toxins was recently reported in a tetanus study. Scott *et al.* characterized scFvs to TeNT and found that certain scFv combinations exhibited synergistic binding based on competitive binding studies. These findings agreed with their results that some scFvs reduced toxin activity without recognizing the Hc domain, which is known to attach to gangliosides. Thus, the authors suggested that the synergy in binding stems from conformational changes in the toxin upon binding of one scFv that favors binding of the next and that the targeted epitope may be less important when identifying toxin-neutralizing MAbs [84].

In accordance with these findings, Yousefi *et al.* detected synergistic neutralizing effects of one pair of MAbs, but not other pairs, that recognized two distinct but very close or overlapping epitopes within the GT1b-binding fragment of TeNT. This pair of MAbs displayed an *in vivo* potency several times higher than the commercial TIG antibody preparation [52]. The authors speculated that the observed cooperative binding effects were caused by conformational changes in the TeNT protein in which binding of the first antibody thermodynamically facilitated the binding of the second antibody.

Ngundi *et al.* investigated the mechanism involved in the synergistic neutralizing effects of combined anti-PA MAbs against anthrax. The authors found that one MAb promoted the bivalent binding of another MAb to PA, presumably by bridging two PA monomers, and proposed that bridging via one MAb promotes bridging via the other. Thus, any transient dissociation of one antibody arm from the toxin would result in rapid rebinding, as the other antibody bridge would prevent the antigen from diffusing away. This phenomenon would substantially increase antibody avidity, *i.e.*, the combined strength of multiple bonding interactions, resulting in synergistic neutralization [46].

All of the evidence described above suggests that the increased affinity of MAb cocktails might result in a synergistic enhancement of toxin neutralization.

### 2.3. Fc-Mediated Clearance of Antibody-Toxin Complexes via Multimeric Antibody Decoration of the Toxin

Low-affinity FcRs do not bind to monomeric immunoglobulins at a detectable affinity; nevertheless, they bind to aggregated antibodies complexed to multivalent antigens with a high avidity. Thus, a multivalent Fc-decorated antigen, efficiently recognized by FcγR, is the basis for the effective clearance of ICs rather than unbound antibody or monomeric antibody-antigen ICs [90]. Low affinity FcRs on resident macrophages may be more relevant than are high affinity FcRs with respect to the clearance of ICs [91]. In human liver, low affinity FcγRII and FcγRIII were detected on both Kupffer cells and liver sinusoidal endothelial cells, whereas high affinity FcγRI is not expressed in liver macrophages [92,93]. Additionally, it was demonstrated that the rate of removal of complexes to the liver is proportional to the size of the complex [94].

Consequently, the adherence of individual MAbs to an antigen (such as a toxin) would be expected to form relatively small ICs because one antigen molecule can bind to no more than one MAb (except for rare instances involving repetitive epitopes). Because larger IgG-toxin ICs are much more efficiently cleared from the bloodstream [90,94], it is anticipated that by combining two or more MAbs that bind to distinct toxin epitopes, thus mimicking a polyclonal reaction, larger ICs are formed and the clearance of combined neutralizing MAbs is substantially enhanced. Thus, toxin clearance is considered to be much more effective for a MAb combination than for a single MAb treatment.

#### 2.3.1. Fc-Mediated Serum Clearance of ICs

ICs are formed when soluble IgGs bind to a target antigen. ICs, when directly administered to non-immunized animals or formed following the administration of low doses of antigens to immunized animals, are removed rapidly in part by the reticuloendothelial system (fixed mononuclear phagocyte system) [95] and by certain circulating leukocytes that express receptors for the ICs [94]. Generally, the clearance of ICs following a polyclonal response occurs via two pathways, both of which involve antibody Fc. One clearing mechanism is mediated by Fcγ-receptor (FcγR) binding. The second mechanism is mediated by the complement system, which plays a crucial role in IC handling [96]. These two mechanisms can act independently [97]. Thus, antibody Fc-mediated clearance is thought to play an important role in toxin neutralization, as well. Accordingly, it has been demonstrated that the association between PAbs and BoNT leads to enhanced clearance from the circulation of rats, particularly to the liver and spleen [98].

Notably, other roles of the IgG Fc might obscure its involvement in toxin-IgG IC clearance. For instance, Fc substantially contributes to IgG stabilization in the bloodstream primarily due to protection by the neonatal FcR (FcRn). FcRn plays a critical role in regulating the levels of circulating IgGs in rodents and higher species via strict pH-dependent IgG/FcRn binding [99]. The FcRn-bound antibody is thought to be protected from degradation, thereby increasing the half-life of IgG in serum. Therefore, the use of antitoxin agents that do not bear Fc (such as Fab, scFv, *etc.*) and thus display a shorter half-life than intact IgG would be expected to exhibit reduced efficacy due to a rapid decrease in the concentration of the antitoxin agent in the bloodstream, as demonstrated by BoNT toxicity studies [22,70]. Nevertheless, in addition to the inferior potency of the F(ab')_2_ antitoxin, Mazuet *et al.* showed that in spite of its shorter half-life (10-fold), one F(ab')_2_ retained its neutralization efficacy compared with intact IgG. Interestingly, the MAb that was not affected by Fc deletion displayed a much higher affinity than the other MAb [70].

The elimination of foreign Fc from equine antitoxin preparations serves to lower immunogenicity; nevertheless, polyclonal F(ab')_2_-based preparations protect against toxin challenge without activating Fc-mediated effector functions. This protection suggests that a high dose of F(ab')_2_ may compensate for the lack of Fc-mediated mechanisms and may induce neutralization solely via toxin blocking. Furthermore, it is plausible that the addition of human Fc fragment to the equine F(ab')_2_ preparation would improve antitoxin efficacy and reduce the total amount of foreign protein administered to a patient. However, such manipulation is not applicable for polyclonal preparations and may be difficult to evaluate in animal models due to the variance in FcγRs and complement receptors (CRs) among species. Manipulation of Fc fragments is being performed on MAbs, enabling the examination of the role of Fc in neutralization *in vivo*. Recently, the generation and characterization of an FcγR-humanized mouse that lacks all murine FcγRs was described; this model may enable the accurate prediction of the consequences of linking human FcγRs to IgGs for a particular biological response [100]. Recapitulation of the human-specific FcγR expression pattern in this mouse model was used recently to assess the activity of anti-anthrax toxin MAbs containing specific Fc variants that selectively enhanced their affinity for particular human FcγRs [101].

#### 2.3.2. New Evidence for the Role of IgG Fc in Toxin Neutralization

Accumulating evidence from recent years supports the involvement of IgG Fc (for MAbs and PAbs) in toxin neutralization. Vitale *et al.* showed that F(ab')_2_ fragments of an anti-PA MAb were 10- to 100-fold less potent than the complete IgG in neutralizing anthrax toxin *in vitro*, even though they retained equivalent affinity for PA. The addition of FcR-blocking antibodies greatly reduced the activity of this MAb, and the neutralizing activity of mouse, rabbit, and human antisera elicited by PA vaccines was effectively abrogated by blocking FcRs. However, *in vivo*, significantly higher concentrations of F(ab')_2_ did protect against anthrax toxin cytotoxicity; thus, *in vivo* protection cannot be unambiguously attributed to the Fc-enhanced neutralization activity [102]. It is unclear whether this potency reduction *in vivo* is also attributed to the shorter half-life of the F(ab')_2_ fragments. This neutralizing activity was abrogated by FcR blockers for PAb and MAb treatment, implying that their potency is indeed FcR-dependent. Experiments conducted by Arturo Casadevall and colleagues have broadened our understanding of the function of Fc in anti-PA antibodies [103]. Abboud *et al.* generated identical variable regions and specific IgG2a and IgG2b variants of an IgG1 anti-PA MAb and found that the efficacy of antibody-mediated neutralization was dependent on the isotype and that this neutralization activity required a competent FcγR. Furthermore, they showed that the IgG2a MAb prevented lethal toxin cytotoxicity more efficiently than the IgG1 MAb and that passive immunization with IgG1 and IgG2a MAb protected wild-type, but not FcγR-deficient, mice against B. anthracis infection [103]. Nevertheless, the precise toxin neutralization mechanism after FcγR engagement was unclear. The authors hypothesized that MAb PA-FcγRs ICs are endocytosed and sorted to lysosomes for degradation but suggested that the differences in efficacy between the isotypes implied a difference in Fc-mediated effector functions rather than defective endocytosis in mice deficient in FcγRs.

The function of Fc was also evaluated using other anti-toxin systems. Akiyoshi *et al.* evaluated MAbs directed against the A and B subunits of Shiga toxin 2 (Stx2), which is the primary virulence factor for hemolytic uremic syndrome (HUS). The investigators generated and evaluated (in vitro and *in vivo*) isotype variants (IgG1, IgG2, IgG3, and IgG4) and Fab and F(ab')_2_ fragments of a MAb specific for the A subunit of Stx2. These isotype variants exhibited protection *in vitro*, and the IgG3 and IgG4 variants exhibited the highest protection *in vivo*. Although the Fab and F(ab')_2_ fragments exhibited protection *in vitro*, they did not exhibit protection *in vivo*. The authors obtained similar results for a MAb directed against the B subunit of Stx2 [104].

Pincus *et al.* examined the role of the Fc region in mediating protection from ricin toxicity and found comparable results to those for Stx2. They compared the *in vitro* and *in vivo* effects of intact Ig and of Fab fragments derived from PAb and anti-A chain MAb preparations. The results revealed little difference between Ig and Fab in terms of antigen binding and *in vitro* neutralization in HeLa cells but found relatively large differences in their protection of animals. In addition, *in vivo* experiments demonstrated that the murine MAb protected more strongly than its chimeric mouse/human variant, indicating that mouse Fc regions perform better than human Fc in mice. Moreover, the investigators explored the role of FcγR in the protection of cells from ricin toxicity using a panel of HeLa-derived cells carrying no FcR or human FcγRI, FcγRIIa, FcγRIIb, or FcγRIIIa and found that the presence or absence of FcR did not influence MAb-mediated protection. These findings indicated that neither the Fc region nor the presence of an FcR on the target cell influence the Ab-mediated protection of individual cells. However, *in vivo*, the presence of Fc enhanced their protective efficacy [59].

Varshney *et al.* compared the neutralizing and protective activity of anti-SEB MAbs *in vitro* and *in vivo*. The authors demonstrated that changing the isotype of already protective MAbs, without affecting their antigen specificity or sensitivity, enhanced their protective ability, suggesting a therapeutic role for Fc [50].

To directly determine the role of Fc, we recently measured the protective properties of IgG MAb and its F(ab')_2_ form in mice exposed to BoNT/A. Equal amounts of IgG MAb and its F(ab')_2_ fragment (50 pmol/mouse) were incubated with increasing amounts of BoNT/A ranging from 10 to 100 MsLD_50_. Then, these mixtures were injected into mice, and their survival was monitored. The intact IgG MAb protected 100% of the mice from a challenge of up to 50 MsLD_50_ BoNT/A, whereas its F(ab')_2_ fragment protected against only 10 MsLD_50_ of the toxin. Thus, the protective efficacy of F(ab')_2_ in mice was 5-fold lower than that of its IgG form, demonstrating the important role of Fc in protection against BoNT toxicity (paper in preparation).

#### 2.3.3. Synergistic Effect of Oligo-Fc Toxin Decoration

**Figure 4:**
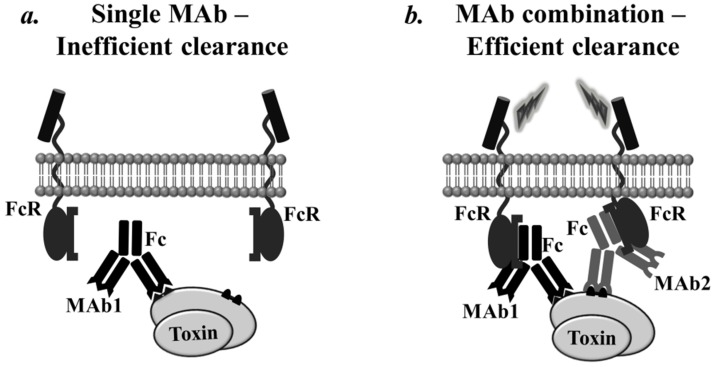
Schematic illustration of the Fc-mediated clearance of antibody-toxin complexes via multimeric antibody decoration of the toxin. The clearance of ICs occurs via FcR and CR pathways, both involve antibody Fc. Low-affinity FcRs on resident macrophages bind to antibody-antigen complexes with a high avidity but do not bind to monomeric antibody-antigen ICs at a detectable affinity. In addition, larger IgG-toxin ICs are much more efficiently cleared from the bloodstream. Thus, a MAb cocktail might mimic a polyclonal reaction by forming large ICs with multiple Fc toxin decoration, resulting in a substantial enhanced antibody-toxin complexes clearance. The figure illustrates binding of multiple Fcs with high avidity to FcRs (**b**), as opposed to low affinity binding of a single MAb Fc (**a**).

Recently, several studies conducted by the lab of Charles Shoemaker directly investigated the phenomenon of synergistic neutralization mediated by increased Fc valence (illustrated in Figure 4). Sepuldeva *et al.* proposed a novel concept in which BoNT neutralization is exerted by combinations of high-affinity epitope-tagged scFvs against BoNT/A, serving as the ‘blocking’ arm, and anti-tag MAbs containing Fc, serving as the ‘clearing Fc’ arm. In this manner, the investigators elegantly illuminated the critical contribution of Fc-mediated clearance of BoNTs to synergistic neutralization and dissected the function of Fc from other putative mechanisms. The authors showed that for individual non-neutralizing scFvs, the addition of additional clearing Fcs synergistically increased the level of protection against BoNT/A. Thus, when an scFv contained two copies of the tag (*i.e.*, two ‘clearing Fcs’ for each ‘blocking arm’) instead of one, protection against the toxin was approximately 10-fold enhanced, approximately the same fold-change as the addition of a different scFv. The improvement achieved by the inclusion of an additional tag likely resulted from further Fc decoration of the toxin. Following that study, the definitive role of Fc-mediated clearance in synergistic activity was demonstrated by pharmacokinetic studies showing that BoNT/A was rapidly cleared from the sera of mice administered a pool of anti-BoNT/A scFvs and a clearing Fc but not from the sera of mice administered either the scFvs or the clearing Fc alone. Moreover, the efficacy and clearance profile of this mixture of a neutralizing dose of scFvs combined with an anti-tag clearing Fc was comparable to that of a polyclonal antitoxin serum [24].

Additional experiments were performed by the same lab to further investigate the role of toxin blocking and clearance in determining antitoxin efficacy. The authors generated small (12 kDa) and stable camelid heavy-chain-only Ab VH (VHH) domains against BoNT/A. These VHHs were used as antitoxins via the co-administration of recombinant high-affinity anti-BoNT tagged VHH and an anti-tag clearing Fc. The authors compared neutralizing or non-neutralizing binding VHHs administered with or without clearing Fc to evaluate the relative contributions of toxin blocking and clearance to antitoxin activity. A cocktail of up to three VHHs, each containing one tag, administered with an anti-tag clearing Fc was insufficient to induce efficient clearance. However, a heterodimer consisting of two non-neutralizing monomeric VHH units, each containing two tags instead of one, enabled the simultaneous binding of four Fc domains to the toxin. The resulting toxin clearance dramatically enhanced the protection of mice from virtually no protection in the absence of the clearing Fc to complete protection against 1000 MsLD_50_ of BoNT/A. Furthermore, a comparison of the antitoxin efficacy between neutralizing and non-neutralizing VHH agents in the presence or absence of the anti-tag clearing Fc revealed that the neutralizing VHHs were highly effective in the absence of clearance (and were improved in the presence of the clearing Fc), whereas non-neutralizing VHHs depended on the clearing Fc for efficient neutralization [23].

A similar technique utilizing a VHH-based neutralizing agent (VNA) and a clearing Fc agent was demonstrated to be effective for Stx. Tremblay *et al.* showed that co-administration of a VNA and an anti-tag Fc substantially increased the prevention of death or kidney damage in mice following challenge with Stx1 or Stx2 [105].

However, in a recently conducted anti-ricin study, contradicting results were obtained. Vance *et al.* showed that on one hand, the addition of a clearing Fc agent to double-tagged VHH heterodimers (enabling up to four Fc molecules to decorate each toxin molecule) significantly increased the protection of mice from ricin toxicity, likely via the promotion of toxin clearance [106]. On the other hand, the presence or absence of an anti-tag clearing Fc linked to a high affinity heterodimer consisting of two non-neutralizing VHHs afforded the mice no protection against ricin challenge. Because ricin is toxic to all cell types and preferentially targets macrophages, including Kuppfer cells in the liver [68], the authors postulated that the accelerated FcγR-mediated clearance of ricin in the absence of the inhibition of functional ricin domains may not improve the clinical results and might even enhance its toxicity [106].

The significance of Fc valence for toxin clearance from the circulation was recently shown using red blood cells (RBCs) mediated toxin sequestration [28]. Opsonization of particulate pathogens by antibodies and complement can lead to their binding to the complement receptor1 (CR1), specific for C3b, on primate erythrocytes. This immune adherence may prevent pathogens from leaving the bloodstream and facilitate their destruction by liver macrophages [107]. Ronald Taylor and colleagues used MAbs specific for CR1 cross-linked with pathogen specific MAbs to generate heteropolymers (HPs) which can bind a wide range of substrates to primate erythrocytes, and imitate the natural clearance mechanism of C3b-opsonized substrates bound to erythrocyte CR1. In this way, Fc receptors on the phagocytic cell engage the erythrocyte-bound complex, CR1 is removed by proteolysis, and the entire immune complex and CR1 are internalized while sparing the erythrocyte [107]. The HP methodology was recently applied by Sharma *et al.* that used BoNT as a model system in an attempt to enhance toxin rather than particulate pathogen neutralization. They converted a pair of BoNT specific MAbs into HPs and tested them in transgenic mice expressing human CR1 on RBC membranes. The authors reported that two HPs given in combination had 166-fold greater potency than un-modified MAbs, capable of neutralizing 5000 MsLD_50_ BoNT/A. The ICs formed with an HP and an un-modified MAb were less potent than those formed with two HPs. In addition, peritoneal macrophages internalized BoNT better when it was bound to two HPs rather than to an HP + MAb or MAb + MAb combination, independent of whether the HP pair contained a CR1-binding or nonbinding control MAb. This may indicate that enhanced BoNT clearance from the blood circulation by fixed tissue macrophages is attributed to the opsonization of multiple Fc domains in the HP complexes [28].

## 3. Conclusions

Currently, modern biotechnological advancements have enabled the manufacture of an unlimited supply of human or humanized MAbs, as well as other mono-specific biopharmaceuticals, that display exquisite specificity and unprecedented affinity for life-threatening toxins and that exhibit a low risk of immunogenicity, low batch-to-batch variability, and a long serum half-life of up to 1 month, reducing the frequency of drug administration [9,21,58].

These characteristics are highly valuable and advantageous compared with PAb-based preparations. However, the antigenic variability of toxins (and pathogens in general) and the relatively low efficacy of individual antitoxin MAbs limit their utility as therapeutics. One rational solution to overcome these limitations would be to combine MAbs into therapeutic cocktails to mimic safe polyclonal responses. Until recently, this option was difficult to implement due to regulatory and cost concerns [47]. Nevertheless, efforts in recent years to produce effective MAb cocktails for post-exposure rabies and botulinum prophylaxis [15,108] have established an important precedent by increasing the relevance of the use of MAb combinations against toxins and infectious diseases.

Many studies have shown (Table 1) that different combinations of MAbs against various toxins and other infectious agents indeed exhibit enhanced efficacy and that in many cases, this enhancement is synergistic, occasionally exceeding the efficacy of the PAb-based preparations that are generally used. In this review, we described a body of evidence supporting three potential mechanisms underlying synergistic neutralization by MAb combinations: (1) the simultaneous binding of interfering MAbs to multiple functional sites of the toxin, facilitating the significant blockade of two or more essential epitopes that react with the target cell, thus dramatically reducing the potency of the toxin; (2) a dramatic increase in the affinity of the combination of MAbs compared with each MAb component, correlating with neutralization; and (3) Fc-mediated clearance of antibody-toxin ICs from the serum, which is enhanced by the multimeric antibody Fc decoration of the toxin. It is plausible that these three mechanisms complement each other and that the combination of these mechanisms mediates the enhanced cooperative activity of MAb cocktails.

These mechanisms may also clarify the contribution of individual non-neutralizing MAbs to the synergistic neutralization of a MAb cocktail, a phenomenon that was observed in previous studies by our lab and by others [16,50,51,71]. This synergism can be facilitated by increasing the valence of Fc molecules decorating the toxin, thereby enhancing toxin clearance, by targeting a critical number of epitopes, by increasing the avidity of the MAb cocktail, or via the interaction of two or more mechanisms. One conclusion may be that screening for toxin-neutralizing MAbs should involve high-affinity MAb combinations to avoid overlooking MAbs that individually display low or no neutralizing activity.

MAb cocktail-based drugs that display enhanced neutralization efficacy can significantly lower the amount of drug administered in comparison with the efficacy of a single monoclonal antibody, which will likely reduce cost and side effects.

The synergistic activity of MAb combinations may depend on the presence of Fc and on the Fc isotype [46,103,109]. Thus, an *in vitro* assessment of the neutralizing capacity of a MAb combination might not fully reflect its *in vivo* potency. Moreover, the evaluation of a MAb from a certain animal origin might not exert its full potency due to the inter-species variance of FcRs and CRs. Therefore, special care should be taken when selecting the animal model for evaluating the synergistic effect of a MAb combination.

Understanding the mechanisms underlying synergistic neutralization is crucial for combining MAbs to form potent antibody cocktails. Future experiments should focus on developing analytical tools that will enable further investigation of these mechanisms. It is tempting to speculate that control of the enhancement of neutralization might become feasible via the rational selection and combination of MAbs.
